# An Integrated Artificial Intelligence of Things Environment for River Flood Prevention

**DOI:** 10.3390/s22239485

**Published:** 2022-12-05

**Authors:** Zakaria Boulouard, Mariyam Ouaissa, Mariya Ouaissa, Farhan Siddiqui, Mutiq Almutiq, Moez Krichen

**Affiliations:** 1 LIM, Hassan II University of Casablanca, Casablanca 20000, Morocco; 2 Department of Computer Science, Moulay Ismail University, Meknes 50050, Morocco; 3 Data Science Department, NED University of Engineering and Technology, Karachi 75270, Pakistan; 4 Department of Management Information Systems and Production Management, College of Business and Economics, Qassim University, P.O. Box 6640, Buraidah 51452, Saudi Arabia; 5 FCSIT, Al-Baha University, Al-Baha 65528, Saudi Arabia; 6 ReDCAD Laboratory, University of Sfax, Sfax 3038, Tunisia

**Keywords:** river flood prediction, sensor nodes, lorawan, artificial intelligence, internet of things, artificial intelligence of things

## Abstract

River floods are listed among the natural disasters that can directly influence different aspects of life, ranging from human lives, to economy, infrastructure, agriculture, etc. Organizations are investing heavily in research to find more efficient approaches to prevent them. The Artificial Intelligence of Things (AIoT) is a recent concept that combines the best of both Artificial Intelligence and Internet of Things, and has already demonstrated its capabilities in different fields. In this paper, we introduce an AIoT architecture where river flood sensors, in each region, can transmit their data via the LoRaWAN to their closest local broadcast center. The latter will relay the collected data via 4G/5G to a centralized cloud server that will analyze the data, predict the status of the rivers countrywide using an efficient Artificial Intelligence approach, and thus, help prevent eventual floods. This approach has proven its efficiency at every level. On the one hand, the LoRaWAN-based communication between sensor nodes and broadcast centers has provided a lower energy consumption and a wider range. On the other hand, the Artificial Intelligence-based data analysis has provided better river flood predictions.

## 1. Introduction

Human beings have always tended to live and prosper beside rivers. Iconic civilizations, such as the Pharaohs and the Babylonians, were known for thriving on the banks of the Nile and the Euphrates. These civilizations and others, up until nowadays, have always been under the threat of river flooding, which can have disastrous effects, ranging from taking human lives to destroying both agriculture and infrastructure, thus, causing repetitive economic crises. The extreme climate changes that occurred during the 20th century and are still occurring during the first quarter of the 21st have increased the frequency and magnitude of floods. According to the European Commission [1], river floods are the most common natural disaster in Europe, with annual damages of €11 billion (€98 billion worldwide). Different causes are behind river floods, whether they be other natural disasters, such as heavy rainfalls and hurricanes, or human intervention, such as urbanization, deforestation, and mining [2]. The human impact has even caused changes in flooding behaviors. In Western China, the Weihe River is known to flood during summer and autumn, but sudden land use changes and the industrialization of the area have caused radical changes in the river run-off [3]. The same can be said for a shift in flooding patterns in the Mekong Delta of Vietnam, which is considered the third largest delta in the world. Such shifts may heavily impact policy decisions regarding different issues such as water management, urbanization, agriculture, etc.

Heavy flooding has a huge impact on different socioeconomic fields. For instance, in agriculture, it can cause drastic policy adjustments. A study by Wang et al. [4] put forward the impact of the Yangtze River floods in 2020 on China’s importation of feeds and grains from the USA, raising its rates to over 316% in the first semester of 2021. In Uganda, Mubialiwo et al. [5] presented a model that estimated the direct losses due to the recurrent Malaba river flooding as $33 Million in a two-year return period, including over $855,000 in worshipping places alone. Other socioeconomic infrastructures remain under direct flood threat, including 27 health and education facilities. In Brazil, Silva Araújo et al. [6] assessed the correlation between income inequality and the vulnerability to flooding risks in the Itapocu river basin area. Their study showed that low-income households tend be positioned in riskier areas, thus are more susceptible, than their higher income counterparts, to having their homes and farms destroyed by floods. As an effect, the Itapocu river floods are regarded as an important cause of socioeconomic inequality in its surrounding area.

Governments and research organizations have heavily invested in finding efficient approaches to preventing river floods. The most common method is based on measuring the water level from different locations on a river’s course. Despite its efficiency in normal case scenarios, this traditional method has presented serious shortcomings when it comes to predicting extreme flooding. Some attempts are worth mentioning, such as [7] who suggested a decision support system based on two hydrological models, coupled with a web application for monitoring river levels. Other methods are based on Geographic Information Systems (GIS), as well as images captured by autonomous cars [8,9]. Others attempts were based on analyzing satellite images, such as the work done by Nghia et al. [10] who built a logical model based on the “Google Earth Engine” and “Sentinel 1” data to monitor flood levels downstream of the Mekong River.

One of the main issues that these methods face is the lack of real-time monitoring capabilities that can keep the decision-makers updated on the current issue of the rivers and can predict imminent flooding. In this paper, we will address this issue by presenting an efficient and fully integrated Artificial Intelligence on Things (AIoT) architecture. In this architecture, the sensors placed on the water bodies can communicate the data collected using the LoRaWAN protocol, known for its wide coverage and low energy consumption. The collected data is sent to the closest local broadcasting tower, which will relay the data to the centralized cloud server. This server will process the data based on Artificial Intelligence and will predict the river’s status.

In the next section, we will discuss different approaches in the literature that addressed the flood prediction issue. In Section 3, we will further develop our approach, then discuss the results and findings in Section 4. Section 5 will conclude the paper and suggest different areas of improvement.

## 2. Related Works

Wireless Sensor Networks (WSNs) are a combination of spatially dispatched sensors that monitor real-time physical reactions, such as temperature, motion, vibration, and produce data which will be later collected by sink nodes [11]. Siddique et al. [12] presented an approach based on a WSN that was able to warn nearby villages in case of imminent flooding. They placed four sensors at different locations on the river to constantly monitor its flow and transmit the data to a sink node that will generate a warning in case of a flood. Lukić et al. [13] suggested a WSN architecture where sensor nodes periodically measure different variables such as flow rate, temperature, rainfall, etc. The measurements are compared on sensor levels with two thresholds before being sent to the sink node. Wahyono et al. [14] presented a solution to optimize the performance on a river flood detection WSN by finding the best locations where sensors can be clustered. Their method for grouping the sensors is based on the K-Means algorithm, and the data taken from each grouping is used to predict potential floods in the area it covers. Saharia et al. [15] put forward a WSN architecture consisting of four sensors (ultrasonic, water flow, raindrop, and temperature/humidity), and an Arduino UNO that transmits the collected data to a cloud monitoring system. SMS alerts are sent in case of emergency.

The Internet of Things (IoT), as defined by Oracle [16], is a network of physical objects or “things” embedded to sensors and other devices so they can connect and exchange data with each other via the internet. As of 2020, the number of interconnected IoT devices is estimated to be 10 billion, and it is expected to grow to 22 billion by 2025. Soh et al. [17] presented an architecture where camera-equipped IoT devices capture river water levels and compare them to the riverbank level. When the flood severity reaches a critical level, an alert is sent to the threatened community via social media (Telegram App). Biantoro et al. [18] developed an IoT prototype backed by the Blynk platform [19]. The “FEDS” prototype consists of a microcontroller, a rainfall sensor, and an ultrasonic sensor. Acosta et al. [20] presented an IoT-based solution for flood monitoring. They implemented ultrasonic sensors in 31 interconnected stations along two rivers, and the collected data is consolidated in a cloud platform where an overall dashboard will alert the authorities in case of emergency. Ganesh et al. [21] put forward an IoT-based solution to monitor dam water levels and alert people in case of imminent flooding. They used different sensors to check on the levels in both the dam as a whole and its main pipeline. The collected data is transmitted to a cloud platform.

As defined by [22], Low Power Wide Area Networks (LPWAN) are a set of standards that govern the IoT communication that have specific aspects such as very low battery consumption, wide area coverage, transmission of small data packets, etc. LPWAN-based solutions have been applied in different fields and have displayed great capabilities to connect the IoT devices even in strained circumstances. According to [23], different technologies follow the LPWAN standards but the three most famous are:

- “LoRaWAN”, which is known for transmitting in several sub-gigahertz frequencies, making it less inclined to have interference.
- “Weightless SIG”, which runs in the sub-1 GHz spectrum but can also support 12.5 kHz narrowband technology.
- “NB-IoT”, which can operate on existing cellular infrastructure by taking advantage of the 3GPP standards.

Manuel et al. [24] suggested a novel communication architecture for a search and rescue (SAR) mission. This architecture is based on the LoRaWAN, and a SAR robot called “Rescuer”, which can be deployed in areas where all communication networks are supposed to be wiped out. Ibarreche et al. [25] presented a flash flood prediction and early warning sensor system based on the LoRaWAN architecture. Their “Emergency Water Information Network” revolves around meteorological stations, fixed and mobile water monitoring stations along the river, and a data transmission unit. Sung et al. [26] proposed a LoRaWAN- and SIM900-based mountain flood observation system where the communication between the sensors and a cloud server is ensured via the internet. The data is accessible via a special app, and warnings are sent via SMS. Ragnoli et al. [27] put forward a modular LoRaWAN-based architecture of a flood monitoring system where no major updates in the hardware disposition would be necessary. They wired the sensors to a microcontroller which will send the data through the LoRaWAN to a web structure that will take charge of raising the alarm in case of floods. Yeon et al. [28] suggested a real-time flood monitoring system based on flood sensors communicating through the NB-IoT and transmitting their collected data to the sever using TCP/UDP protocols. Di Gennaro et al. [29] proposed a water monitoring system where the communication between the sensors is based on Weightless Sigfox.

Artificial Intelligence (AI) is a set of algorithms which, based on the rapid growth of data, can efficiently provide computers and machines with the ability to sense their environment, learn from it, and react to it [30]. AI has an incredible impact on different socioeconomic fields, and it is expected by 2030 to add up to $13 trillion to the GDP [31]. AI has demonstrated its capacities in different fields [32,33,34,35,36]. For instance, the authors in [37] suggest a sparse regression-based, data-driven model identification for ships with 6 degrees of freedom. In [38], the authors propose a solution to optimize sensor mapping in smart homes based on deep adversarial transfer learning. In [39], the authors provided a means to optimize the performance of hyperspectral image classification by combining the PCA, LBP, Grey Wolf, and KELM algorithms. In [40], the authors suggest a new approach to solve the problem of airport gate allocations. Their solution is based on a combination of a quantum-inspired co-evolutionary algorithm, along with random rotation direction, and a Hamming adaptive rotation angle. As for river flood monitoring, Hasanuzzaman et al. [41] compared the performance of three Machine Learning (ML) algorithms for flood susceptibility mapping. Their dataset consisted of 500 historical flood points with twelve influencing factors (elevation, rainfall, slope, etc.). The best performance was provided by the Random Forest (RF) algorithm. Luppichini et al. [42] fully relied on historical data when training their Long Short Term Memory (LSTM) algorithm and tried to prove that their approach remains able to provide good flood level predictions, even in case of data shortage. Atashi et al. [43] also relied on historical data, namely, twelve years of hourly data from three stations along the Red River of the North. They used this data to train the RF, LSTM, and Seasonal Autoregressive Integrated Moving Average (SARIMA) algorithms. The LSTM algorithm has provided the best flood level predictions. Tanim et al. [44] put forward an unsupervised Machine Learning approach based on a combination of the Change Detection method, the Otsu algorithm, and fuzzy rules. This approach was tested on flood images captured by the ESA’s “Sentinel 1” satellite and has proved better classification performance when compared to the Random Forest (RF) algorithm, Support Vector Machines (SVM), and the Maximum Likelihood Classifier (MLC). The Artificial Intelligence of Things (AIoT) is an architecture that takes the best of both AI and IoT. It is expected to represent the trend of the future due to its widespread applicability that can cover every possible domain including environment and weather monitoring [45]. Previous literature presents several models using the AIoT as an efficient means of real-time monitoring of river levels, and of providing predictions on eventual river floods. Goudarzi et al. [46] proposed a multilevel architecture where WSNs are backed by Unmanned Aerial Vehicles (UAV) that can act in case of connectivity issues. The collected data is fed to a Particle Swarm Optimization (PSO) algorithm that will predict forthcoming floods. Fernandes Junior et al. [47] suggested a lightweight Deep Neural Network (DNN) that can be embarked in memory-constrained IoT devices deployed for river flood detection. Mangukiya et al. [48] proposed an IoT-based architecture where sensors are dispatched along the lower Narmada basin in India. The collected data is fed to a Random Forest algorithm that was trained on historical data taking different variables into consideration such as elevation, slope, drainage density, annual rainfall, etc. A RandomizedSearchCV algorithm was deployed prior to the RF training for a better performance. Anuradha et al. [49] put forward an LSTM-backed IoT hazard monitoring and prediction system taking into consideration the specific case of hills and mountainous terrain. Their system is meant to cover different natural disaster scenarios such as flooding, landslides, and avalanches.

With the recent advances in microcontroller-related technologies, embedded Machine Learning has gained momentum and demonstrated its capabilities in different areas. For instance, Dudak et al. [50] implemented a neural network model in an STM32L4x microcontroller for motion detection purposes, enabling it to recognize six basic movements in three axes. Healthcare has its fair share of embedded Machine Learning applications. On the one hand, Gokul et al. [51] implemented the ProtoNN embedded classifier [52] in an ATMega2560 microcontroller to detect Freeze of Gait events in Parkinson Disease patients. On the other hand, Peruzzi et al. [53] implemented a CNN model in a microcontroller making it able to detect and prevent sleep bruxism. Incorporating embedded Machine Learning in river flood prevention systems is a recent research area with promising results. For example, Syed Ahmed Ali et al. [54] implemented a Machine Learning model in an ESP32 microcontroller that collects data via 4G from precipitation, water flow, and water level sensors. The alert is sent to the community via an Android app connected to the microcontroller. Coveñas et al. [55] set up a Wireless Sensor Network centered around an ESP32 microcontroller with an implemented Machine Learning model making it able to prevent eventual floods and mudslides. Shun-Nien Yang et al. [56] implemented an IoT-based sensor network enhanced with Machine Learning capabilities to predict the average regional flood depth in the Erren river basin in south Taiwan. Khalaf et al. [57] merged an IoT sensor network with an ensemble learning model combining LSTM and RF to provide an optimized river flood prevention.

Table 1 provides a general overview about the different approaches described in this section.

The studies summarized in Table 1 have one main issue, which is that they base their works on covering one river at a time; this raises several questions regarding scalability, especially with the possibilities offered by the AIoT and Big Data Analytics. In this paper, we introduce a fully integrated AIoT environment that can provide a more efficient insight on the data collected by sensors from different rivers, and thus, can better prevent the possibility of floods. This environment will have a multilayer architecture. In the lowest layer, a Low Power Wide Area Network (LPWAN) will connect different sensors that are widespread over all the rivers. The collected data will be fed to a Deep Learning layer that will analyze the data and predict the status of the rivers countrywide.

## 3. Materials and Methods

### 3.1. LPWAN Architecture

#### 3.1.1. LPWAN

Long distance and low power communication is one of the main challenges for the IoT and M2M. LPWAN networks can face this challenge by adopting a star network architecture and allowing communications of up to 20 kilometers in a free field. This network topology is based on a central piece of equipment that will direct all connections. Each sensor will therefore send messages to a local hub but will not be able to send messages directly to other transmitters. This technology makes it possible to send and receive messages of very small sizes, with the major advantage that the components used to send these messages are very inexpensive and very energy-efficient. As explained by Adefemi et al. [58], it is now possible with a simple battery to send a few messages per day for 10 years.

In addition, LPWAN networks differ from traditional short-range networks such as ZigBee, Bluetooth, and the Wireless Local Area Network (WLAN). The range of these communicating systems, which tops out at around 100 hundred meters, is sometimes compensated for by a denser mesh of gateways or a mesh architecture. This, therefore, implies a much higher network infrastructure installation and maintenance costs. The use cases seem to be closer to the classic uses of the Global System for Mobile communication (GSM) and Long Term Evolution (LTE). Although they have significant coverage, Muteba et al. [59] argued that GSM networks do not compete on the vital points of the IoT, namely consumption and prices.

**Figure 1:**
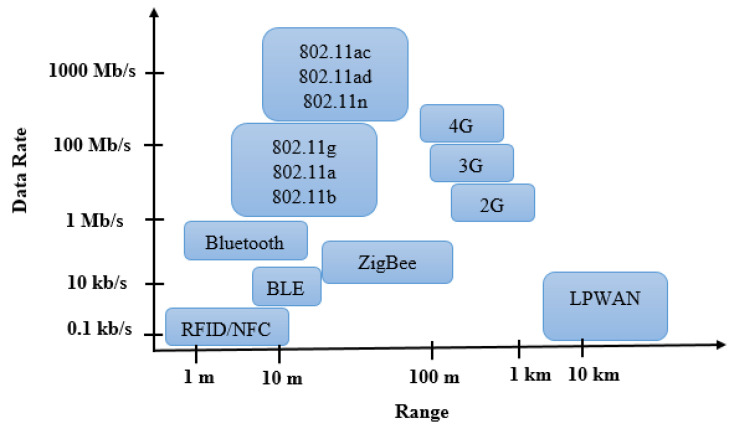
Data Rate vs. Range: LPWAN Positioning.

In Figure 1, we can see where the LPWAN networks are located in relation to the others, in terms of power consumption, flow, and distance. It is interesting to note that they occupy an important place on the graph covered by no other communication protocol.

#### 3.1.2. LoRaWAN

A LoRaWAN (Low Range Wide Aera Network) is a radio communication protocol regulated by the LoRa alliance [60]. It allows the transmission of low amounts of data between different devices. It is based on a star topology in which gateways or hubs relay messages between devices; in our case, sensors and a central data analytics server. As described by Amazon [61], a LoRaWAN allows a communication range of up to 10 miles, and a battery duration of 10 years. It offers a license-free radio spectrum, and its devices are of low cost in either purchasing or maintenance.

The choice of a LoRaWAN in our case scenario of river flood monitoring is based on the different advantages it offers. The first is its ability to transmit small amounts of data in frequent time frames, and with low power needs. The second is its built-in ability to fit into a star-based topology. The third is that, as argued by both Tadrist et al. [62] and Ortega-Gonzalez et al. [63], a LoRaWAN has demonstrated its robustness in adverse weather conditions, which usually go hand-in-hand with river flooding.

#### 3.1.3. The Suggested Architecture

**Figure 2:**
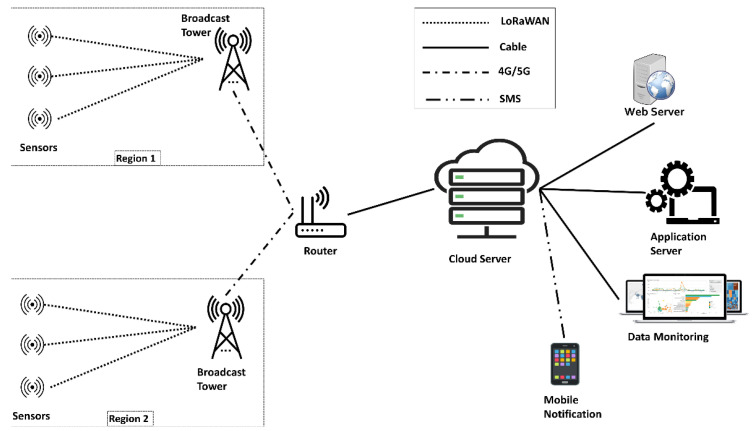
The suggested architecture.

In our architecture, described in Figure 2, we suggest placing flood detection sensors in concerned water bodies. The sensors from each region will transmit their data via a LoRaWAN to their closest broadcast tower, which operates as both a local LPWAN hub and a national 4G/5G transmitter (according to the network protocol adopted by the country). The broadcast tower will transfer the data via 4G or 5G to the cloud server via its router.

The cloud server will analyze the data using an artificial intelligence approach that will be described in the following subsection. The results of the data analytics step will be carried by the application server which will feed them to both a web server and a data monitoring system. In the case of an imminent flood emergency, SMS messages will be sent to the nearby population.

This architecture is using WISE-4610P [64] sensor nodes. This model ensures a data transmission range of 15 km to the closest hub via a selection between either LoRa or LoRaWAN. Its external antenna allows a better obstacle penetration. It offers a built-in 4100 mAh rechargeable battery with a 10~50 V_DC_ external power and a 17–21 V_DC_ solar energy panel. It works on an EU region LoRaWAN frequency range of EU 868 with a data rate of 50 kbps at FSK mode. It has a spreading factor of 7~12, a transmit power of up to +18 dBm, and a receiver sensitivity of −136 dBm at SF = 12/125 KHz. It can operate at a range of −40~85 °C temperature and at a range of 5~95% RH humidity.

The hubs used in this architecture are based on WISE-6610-N100C-A [65] gateways implemented in each broadcast tower. This gateway model offers a support of up to 100 nodes with 951 MHz along with a built-in LTE Cat-M1 cellular interface.

**Figure 3:**
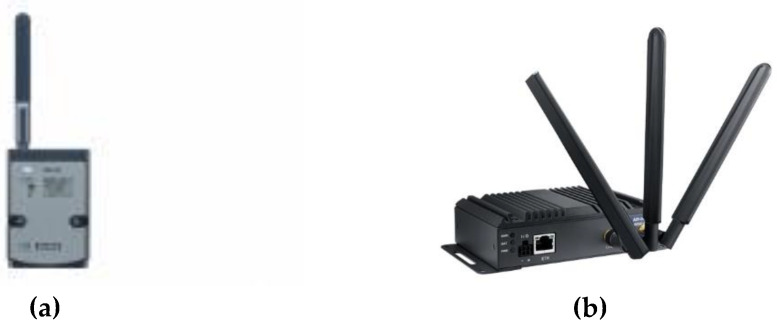
Pictures of the sensor node and hub listed as: (**a**) WISE-4610P sensor node; (**b**) WISE-6610-N100C-A hub [66].

Figure 3 displays pictures of the used sensor nodes and hubs.

When compared to embedded Machine Learning approaches such as the one implemented by Syed Ahmed Ali et al. [54], our architecture, in theory, may gain in performance. In their approach, the authors in [54] used 4G to transmit data from the sensors to the ESP microcontroller. This will consume energy and be a waste of data, since 4G allows the transmission of more data than is needed for a sensor. In our approach, the sensors and hubs communicate via the LoRaWAN. This will consume less energy, especially in sensor level, and take full advantage of LoRaWAN capabilities as being more robust in harsh environments [62].

### 3.2. Artificial Intelligence Approach

#### 3.2.1. England’s DEFRA Catchment Dataset

A suitable dataset for training our candidate artificial intelligence algorithms needs to cover different bodies of water and their flood measurement data, as well as a status variable that can help for the classification. In 2013, the UK’s Department for Environment, Food, and Rural Affairs (DEFRA) launched an initiative called a “Catchment Based Approach” (CBA) [67]. It is a framework that facilitates data collection from different water bodies at local, regional, and national levels by encouraging different actors to take part in the process, whether they be DEFRA officials, industrial collaborators, local politicians, or NGOs.

**Figure 4:**
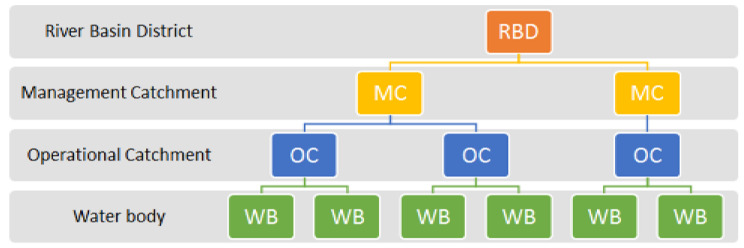
The hierarchy adopted by the Catchment Based Approach [68].

In the CBA, the water bodies are divided into a hierarchical catchment design running from the larger river basin districts to the smaller water body units. The diagram showing the CBA hierarchy is described in Figure 4.

The catchment dataset is provided by DEFRA [68] as open data. It includes flood measurements and classification along with other types of data from the 10 following river basin districts (RBDs):

- Anglian;
- Dee;
- Humber;
- North West;
- Northumbria;
- Severn;
- Solway Tweed;
- South East;
- South West;
- Thames.

These RBDs contain a total of 4950 water bodies grouped into 750 Operational Catchments and 117 Management Catchments.

The dataset, in its latest update on 20 May 2022, contains over 1 M entry with 21 features. The target column “Status” is a multiclass with 13 possible classes. These classes and their value counts are described in Table 1.

#### 3.2.2. Data Preprocessing

Among the 21 features, “Status” is considered as a target, so we needed to isolate it from the rest of the dataset. Other columns, such as “Linked Reasons”, “Ngr”, “Investigation Outcome”, etc, were removed as the columns that either had more than 20% of missing values or too many unique categories were removed. Thus, the number of the most relevant features that were kept is 12.

After that, we checked the number of null values in the dataset. The number of these values was 920,976. We first thought about removing the lines where these data are, but we ended up reducing the size of the dataset from over 1 M lines to a mere 233,770 lines. This drastic reduction would hurt the algorithms’ learning process, so we opted for another option, which is “Forward Filling”. This method will fill every null value with its corresponding value from the previous line. Since there were missing values in the first line itself, a “Backward Filling” was also needed so we can fill the null values with their corresponding counterparts from the next line.

As it can be seen from Table 2, there is a huge difference in the value counts between some of the “Status” classes. For instance, the “Good” class has 330,940 values, while “Upward trend” has only 1022 values. To remedy this difference, we opted to rally these 13 classes into 3 so we could have a better equilibrium.

From those classes, we kept the following 3:

- Good;
- Moderate;
- Bad.

Table 3 displays the old classification values and their matching counterparts in the new classification as well as the value counts of each of the new classes.

Even after resetting the categories, the dataset is still lacking equilibrium. The “Good” class still has more than four times more occurrences than “Moderate”, and around three times more than “Bad”. This imbalance may raise suspicions about the effectiveness of the classification algorithms. To make sure that such a lack of equilibrium would not impact the performance of the candidate algorithms, we run them on two different settings:

- Setting 1: The candidate algorithms run using this data as it is;
- Setting 2: The dataset is rebalanced using the Synthetic Minority Oversampling Technique (SMOTE), a method for oversampling minority classes. To avoid a simple cloning of minority classes, SMOTE is based on a simple principle: to generate new minority classes that resemble the others, without being strictly identical. This makes it possible to increase the density of minority classes in a more homogeneous way.

In this dataset, some of the features such as “Cycle” are numerical, while others such as “Status” and “Water Body Type” are categorical. Ordinal and One-Hot encoding were adopted depending on each of the concerned columns.

#### 3.2.3. Candidate Algorithms

The dataset is a bit structured and contains both numerical and categorical features. According to the literature, Deep Learning algorithms as well as tree-based Machine Learning algorithms tend to perform best in these kinds of scenarios. We chose to compare the performance of four algorithms, two from each category, with our dataset:

- A Decision Tree (DT) algorithm is where the set of choices is represented in the graphic form of a tree, hence its name. A decision tree is a diagram representing the possible outcomes of a series of interconnected choices. It allows a person or organization to evaluate different possible actions in terms of their cost, probability, and benefits. A decision tree typically begins with a node from which several possible outcomes flow. Each of these outcomes leads to other nodes, from which other possibilities emerge. The resulting diagram is similar to the shape of a tree. At the ends of the branches, the leaves of the tree, we find different possible decisions. This type of tool has the advantage of being readable and quick to execute classification tasks.
- A Random Forest (RF) algorithm is composed of several decision trees, trained independently on subsets of the training dataset (bagging method). Each one produces an estimate and it is the combination of the results that will give the final prediction, which results in a reduced variance. In short, it is a matter of drawing inspiration from different opinions, dealing with the same problem, to better understand it. Each model is randomly distributed into subsets of decision trees. In classification, the final estimation consists of choosing the most frequent response category. Rather than using all the results obtained, a selection is made by looking for the most frequent prediction.
- A Deep Neural Network (DNN) algorithm tries to mimic the human brain. The neural network is based on a large number of processors that operate in parallel and are organized in layers. One of these layers receives the raw information inputs, as the optic nerve of the human being operates when it processes visual signals. Then, each layer receives the information output from the previous layer, a process that is found in humans. This is, in fact, what happens during the transmission of information between neurons via synapses, especially near the optic nerve. The results of the system are produced by the last layer of neurons.
- The Long-Short Term Memory (LSTM) is the most widely used recurrent neural network architecture in practice that addresses the gradient vanishing problem. The data transfer process is the same as that of standard recurrent neural networks. However, the information propagation operation is different. As the information passes through, the operation decides which information to process further and which information to discard. The main operation consists of cells and gates. The state of the cell functions as a pathway for the transfer of information. Each cell is composed of three gates which determine whether to let in a new input (input gate), delete the information because it is not important (forget gate), or let it influence the output at the current time step (output gate).

#### 3.2.4. Evaluation Metrics

After preprocessing, we held a comparative study of the performance of the candidate algorithms. The indicators were:

- Accuracy;
- Precision;
- Recall;
- F1-Score;
- Confusion Matrix;
- ROC Curve

*Accuracy* measures the rate of correct predictions for all the values. It is the ratio of the total of true values (True Positives or “*TP*” and True Negatives or “*TN*”) and the overall values, as explained in the following equation:

(1) $$Accuracy=\frac{\left(TP+TN\right)}{TP+FP+TN+FN},$$

“*FP*” being False Positives, and “*FN*” being False Negatives.

*Precision* measures the algorithm’s ability to avoid mistakes while predicting positive values. It is the ratio of the true positives and the overall positives, as explained in the following equation:

(2) $$Precision=\frac{TP}{TP+FP}.$$

*Recall*, also known as “Sensitivity”, measures the algorithm’s ability to get true positives. It is the ratio of true positives and the sum of true positives and false negatives, as explained in the following equation:

(3) $$Recall=\frac{TP}{TP+FN}.$$

The *F*1−*score* evaluates the ability of a classification model to efficiently predict positive values, by making a trade-off between precision and recall. Mathematically, the *F*1−*score* is defined as the harmonic mean of the precision and recall, which translates into the following equation:

(4) $$F1-Score=\frac{2}{\frac{1}{Precision}+\frac{1}{Recall}}.$$

That same equation can be further simplified as following:

(5) $$F1-Score=\frac{TP}{TP+\frac{1}{2}\left(FN+FP\right)}.$$

**Figure 5:**
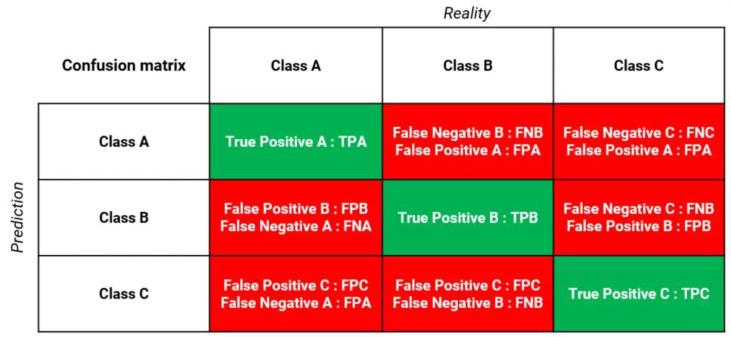
The Confusion Matrix in case of 3 classes [69].

A Confusion Matrix (CM) is a square matrix that displays the number of true positives, true negatives, false positives, and false negative altogether. The matrix lines represent the predicted classes while the columns represent the real classes. In the case of 3 classes, as in Figure 5, the true positives (in green) are in the confusion matrix’s diagonal, while the false positives and false negatives for each class (in red) are outside the confusion matrix’s diagonal.

The receiver operating characteristic curve (ROC curve) is a graph that displays how well a classification model performs across all categorization levels. The True Positive Rate and False Positive Rate are plotted on this curve at various categorization thresholds. The True Positive Rate refers to Recall while the false positive rate is specified as:

(6) $$FPR=\frac{FP}{FP+TN}.$$

## 4. Results and Discussion

As described earlier, the candidate classification algorithms were run under two settings. The first setting used the curated dataset as it is, while the second setting oversampled the minority classes to reach an equilibrium between the three classes. The dataset was split into training and testing subsets with an 80:20 ratio (80% for the training subset against 20% for the testing subset). This choice was based on the argument of Roshan Joseph [70], stipulating that this split ratio tends to provide the best results. The training parameters for the candidate algorithms were tuned using a Random Search cross-validation approach [71] for optimized performance. The results in both settings were evaluated based on the abovementioned evaluation metrics.

**Figure 6:**
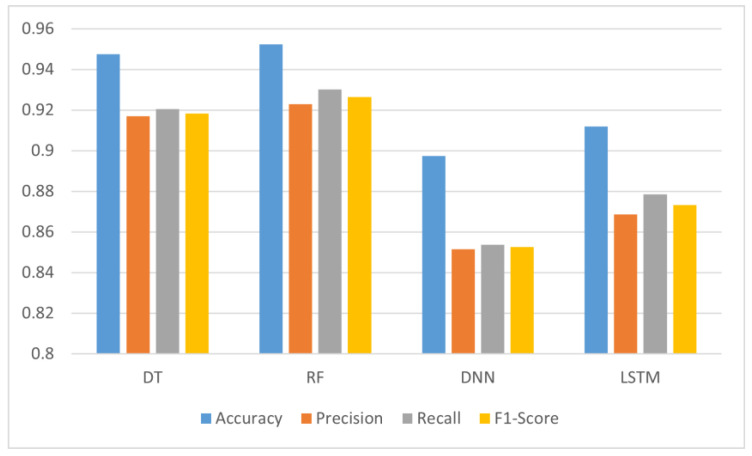
Values of the evaluation metrics for each candidate algorithm in setting 1.

**Figure 7:**
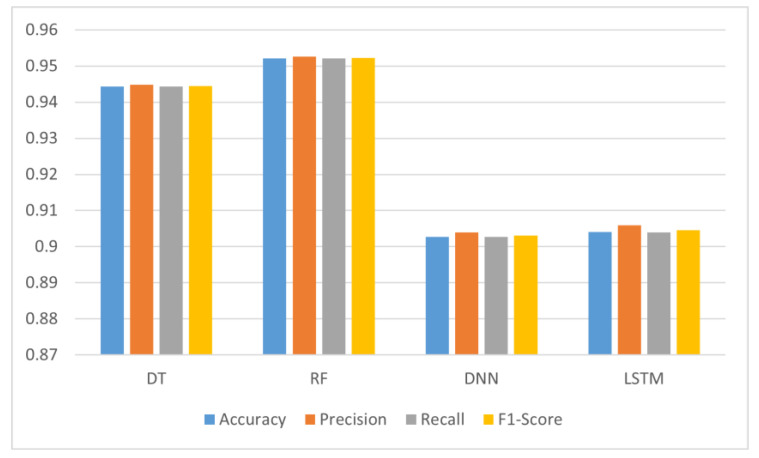
Values of the evaluation metrics for each candidate algorithm in setting 2.

The results, as described in Table 4, and in Figure 6 and Figure 7, display a large advantage in the accuracy of the tree-based algorithms. Indeed, the DT has scored 94.74% in setting 1 and 94.44% in setting 2, while the RF has scored 95.24% in setting 1 and 95.21% in setting 2. On the other hand, the DNN has scored an accuracy of 89.74% in setting 1 and 90.27% in setting 2, while the LSTM scored 91.18% in setting 1 and 90.41% in setting 2.

Accuracy-wise, it seems that the dataset imbalance issue did not affect the performance of the algorithms in general. The only case scenario where balanced data provided more accuracy was with DNN, giving it a 0.01 increase. For RF, the accuracy remained almost the same (a 0.03% decrease). The decrease in accuracy was a bit more important in DT (a 0.3% decrease), and in LSTM (a 0.77% decrease).

**Figure 8:**
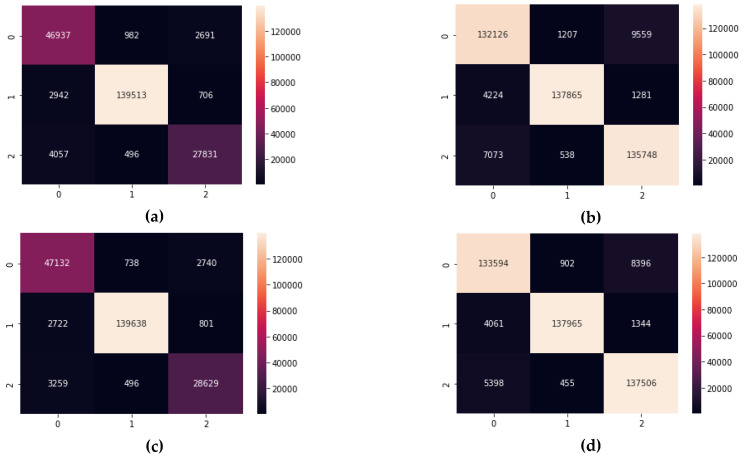
The Confusion Matrices for the tree-based algorithms, listed as: (**a**) DT in setting 1; (**b**) DT in setting 2; (**c**) RF in setting 1; (**d**) RF in setting 2.

**Figure 9:**
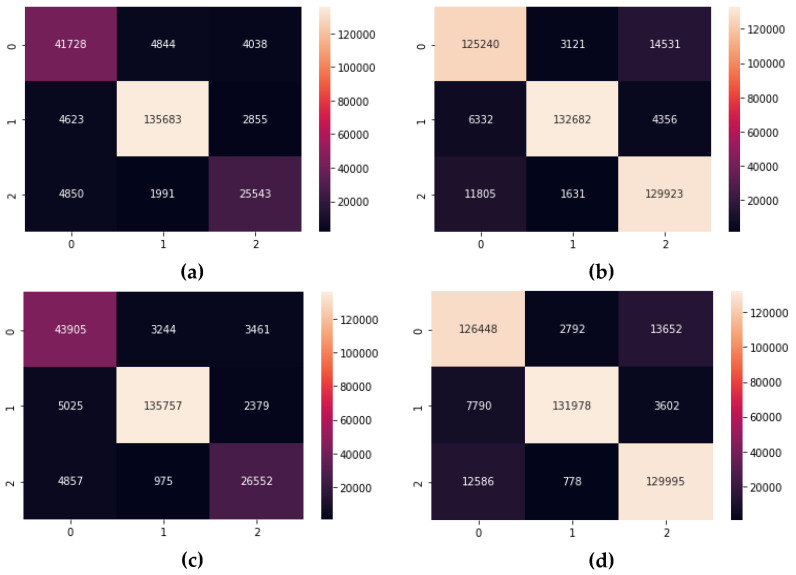
The Confusion Matrices for the Deep Learning algorithms, listed as: (**a**) DNN in setting 1; (**b**) DNN in setting 2; (**c**) LSTM in setting 1; (**d**) LSTM in setting 2.

However, the impact of data imbalance was more visible on Precision, Recall, and the F1-Score, giving them an average increase of 3% for DT and RF, 5% for DNN, and 4% for LSTM. Although, these differences may seem to be low, their effect is more visible on the confusion matrices of each of the candidate algorithms. As displayed in Figure 8 and Figure 9, the number of true positives for all the candidate algorithms is more balanced in setting 2 when compared to setting 1.

**Figure 10:**
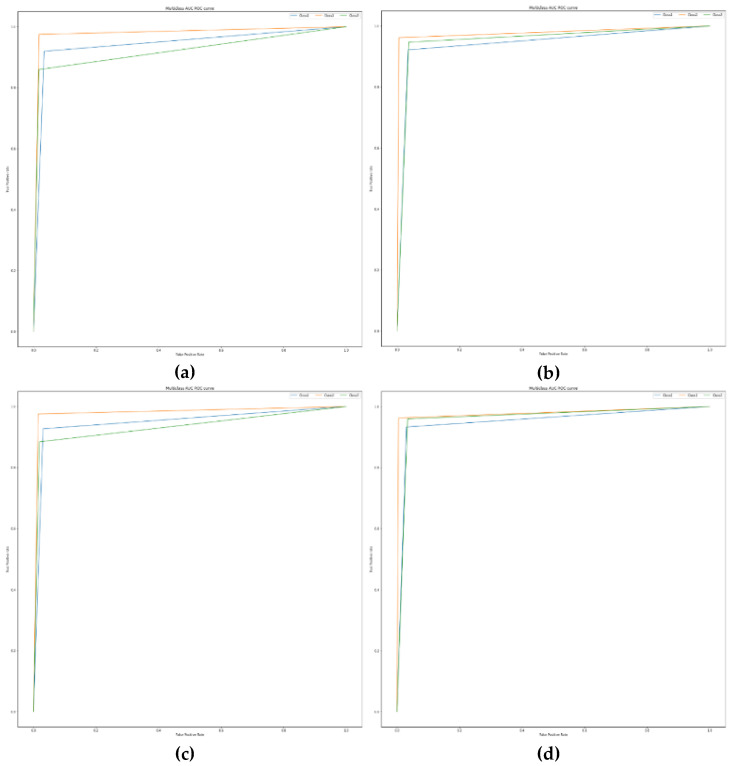
The AUC-ROC curves for the tree-based algorithms, listed as: (**a**) DT in setting 1; (**b**) DT in setting 2; (**c**) RF in setting 1; (**d**) RF in setting 2.

**Figure 11:**
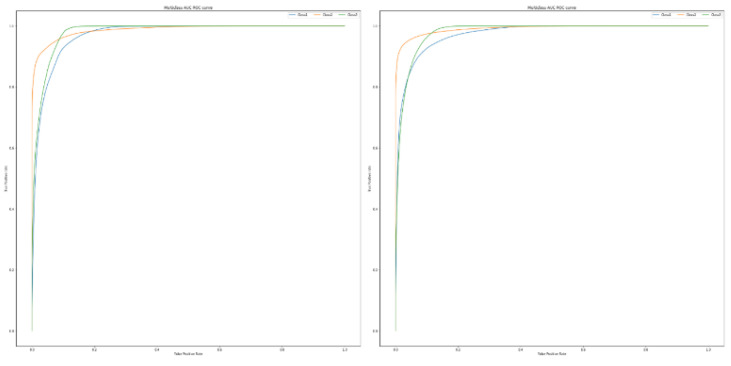

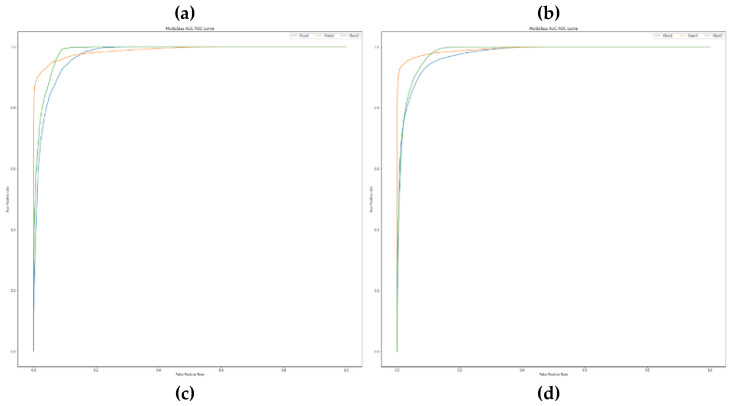
The AUC-ROC curves for the Deep Learning algorithms, listed as: (**a**) DNN in setting 1; (**b**) DNN in setting 2; (**c**) LSTM in setting 1; (**d**) LSTM in setting 2.

Again, and as described in Figure 10 and Figure 11, the effect of class imbalance is evident in the AUC-ROC curve, especially in the case of the Random Forest and Decision Tree algorithms. We can clearly see that in the setting 1, the difference in true positive rate for a particular value of false positive rate is more as compared to that in the setting 2. Additionally, the curves for the Random Forest and Decision Tree algorithms are sharper compared to that of DNN and LSTM signifying that the former perform significantly better than the latter in this case.

As displayed in Table 5, when compared to other approaches that used Accuracy as a performance metric, our approach demonstrated the best accuracy with 95.24%, closely followed by Wahyono et al. [14] with 94%. The approach of Khalaf et al. [57] was third with 85.9% accuracy. The remaining approaches [26,41,44] all had an 85% accuracy.

## 5. Conclusions

This work presented an AIoT based architecture for effective data collection, analysis, and the prevention of imminent flooding that can cover multiple rivers countrywide. This architecture has three levels. The first level contains different sensors that continuously collect data and transfer them to their regional master nodes. The second level of this architecture contains an LPWAN infrastructure. This relays the data collected by the master nodes to the third layer, which contains a centralized Data Analytics server equipped with a Machine Learning-based control system which processes the incoming data and judges whether to signal an imminent flood warning. The Random Forest algorithm has displayed its ability as a best fit for this task with a 95.24% accuracy, closely followed by the Decision Tree algorithm with 94.74%. Deep Learning-based algorithms did not perform as well with an accuracy close to 90%.

Future work will focus on increasing the performance of the AIoT architecture, in both the sensor layer and the data analytics layer. In the sensor layer, we will explore the possibility of lowering energy consumption even further, especially for the data relaying tasks. We will also explore the possibilities provided with embedded Machine Learning, by implementing a Machine Learning model inside a microcontroller that can equip each local hub. In the data analytics layer, we can adopt a federated learning approach where the central data server can relay the learnt models back and forth between the different local hubs, making them learn from each other, and thus, increasing their efficiency.

## Figures and Tables

**Table 1:** Summary table of the literature review.

Adopted Technology	References	Key Performance Indicators
WSN	Siddique et al. [12]	Observation every 30 min
	Lukić et al. [13]	Observation every 15 min RMSE average of 0.5 in most of the sensors
	Wahyono et al. [14]	Accuracy 94%
	Saharia et al. [15]	Real-time data capturing
IoT	Soh et al. [17]	Edge detection
	Biantoro et al. [18]	Error value of about 3%
	Acosta et al. [20]	Real-time data capturing
	Ganesh et al. [21]	Observation every 24 h
LPWAN	Manuel et al. [24]	Communication range of 1.6 mile
	Ibarreche et al. [25]	Real-time water level monitoring
	Sung et al. [26]	Observation every 10 min Accuracy 85%
	Ragnoli et al. [27]	Real-time water level monitoring
	Yeon et al. [28]	Real-time water level monitoring
	Di Gennaro et al. [29]	Real-time water quality monitoring
Artificial Intelligence	Hasanuzzaman et al. [41]	Accuracy 85%
	Luppichini et al. [42]	Observation every 15 min Absolute error rates between 5% and 20%
	Atashi et al. [43]	RMSE for Pembina 0.190 RMSE for Drayton 0.151 RMSE for Grand Forks 0.107
	Tanim et al. [44]	Accuracy 85%
AIoT	Goudarzi et al. [46]	RMSE 0.167
	Fernandes Junior et al. [47]	Intersection over Union 0.9505
	Mangukiya et al. [48]	Real-time data capturing
	Anuradha et al. [49]	Real-time data capturing
Embedded ML	Syed Ahmed Ali et al. [54]	Real-time data capturing
	Coveñas et al. [55]	Radio range of 1 Km
	Shun-Nien Yang et al. [56]	RMSE 0.036
	Khalaf et al. [57]	Accuracy 85.9%

**Table 2:** “Status” column classes and their value counts.

Class	Value Counts
Good	330,940
High	291,680
Moderate	160,822
Does not require assessment	131,383
Supports Good	91,336
Poor	62,219
Fail	28,643
Moderate or less	14,361
Bad	9869
Does Not Support Good	6113
No trend	1329
Active	1056
Upward trend	1022

**Table 3:** The “Status” column’s old and new classes and the value counts of the new ones.

Old Classes	New Classes	Value Counts of the New Classes
Good	Good	716,034
High		
Supports Good		
Upward trend		
Active		
Moderate	Moderate	162,151
No trend		
Bad	Bad	252,588
Poor		
Fail		
Does Not Support Good		
Moderate or less		
Does not require assessment		
No trend		

**Table 4:** Values of the evaluation metrics for each candidate algorithm in both settings.

Candidate Algorithm	Evaluation Metric	Values in Setting 1	Values in Setting 2
DT	Accuracy	0.9474	0.9444
	Precision	0.9169	0.9449
	Recall	0.9204	0.9443
	F1-Score	0.9183	0.9445
RF	Accuracy	0.9524	0.9521
	Precision	0.9228	0.9526
	Recall	0.9302	0.9521
	F1-Score	0.9263	0.9522
DNN	Accuracy	0.8974	0.9027
	Precision	0.8515	0.9040
	Recall	0.8536	0.9027
	F1-Score	0.8525	0.9031
LSTM	Accuracy	0.9118	0.9041
	Precision	0.8686	0.9059
	Recall	0.8785	0.9040
	F1-Score	0.8732	0.9046

**Table 5:** Comparing our approach with those that used Accuracy as a metric.

Approach	Accuracy
Our approach	95.24%
Wahyono et al. [14]	94%
Khalaf et al. [57]	85.9%
Sung et al. [26]	85%
Hasanuzzaman et al. [41]	85%
Tanim et al. [44]	85%
